# Feasibility and effectiveness of self-monitoring of blood glucose among insulin-dependent patients with type 2 diabetes: open randomized control trial in three rural districts in Rwanda

**DOI:** 10.1186/s12902-022-01162-9

**Published:** 2022-10-08

**Authors:** Loise Ng’ang’a, Gedeon Ngoga, Symaque Dusabeyezu, Bethany L. Hedt-Gauthier, Emmanuel Harerimana, Simon Pierre Niyonsenga, Charlotte M. Bavuma, Gene Bukhman, Alma J. Adler, Fredrick Kateera, Paul H. Park

**Affiliations:** 1 Partners In Health-Rwanda, Kigali, Rwanda; 2 Non-Communicable Diseases Division, Rwanda Biomedical Centre, Kigali, Rwanda; 3 NCD Synergies, Partners In Health, Boston, MA USA; 4 Global Health and Social Medicine, Harvard Medical School, Boston, MA USA; 5 Kigali University Teaching Hospital, College of Medicine and Health Sciences, University of Rwanda, Kigali, Rwanda; 6 Division of Global Health Equity, Brigham and Women’s Hospital, Boston, MA USA

**Keywords:** Self-monitoring of blood glucose, Diabetes Mellitus, Glycemic control, Sub-Saharan Africa, Feasibility

## Abstract

**Background:**

The prevalence of type 2 diabetes in sub Saharan Africa (SSA) has been on the rise. Effective control of blood glucose is key towards reducing the risk of diabetes complications. Findings mainly from high-income countries have demonstrated the effectiveness of self-monitoring of blood-glucose (SMBG) in controlling blood glucose levels. However, there are limited studies describing the implementation of SMBG in rural SSA. This study explores the feasibility and effectiveness of implementing SMBG among patients diagnosed with insulin-dependent type 2 diabetes in rural Rwanda.

**Methods:**

Participants were randomized into intervention (*n* = 42) and control (*n* = 38) groups. The intervention group received a glucose-meter, blood test-strips, log-book, waste management box and training on SMBG in addition to usual care. The control group continued with their usual care consisting of, routine monthly medical consultation and health education. The primary outcomes were adherence to the implementation of SMBG (testing schedule and recording data in the log-book) and change in hemoglobin A1c. Descriptive statistics and a paired t-test were used to analyze the primary outcomes.

**Results:**

In both the intervention and control arms, majority of the participants were female (59.5% vs 52.6%) and married (71.4% vs 73.7%). Most had at most a primary level education (83.3% vs. 89.4%) and were farmers (54.8% vs. 50.0%). Among those in the intervention group, 63.4% showed good adherence to implementing SMBG based on the number of tests recorded in the glucose meter. Only 20.3% demonstrated accurate recording of the glucose level tests in log-books. The mean difference of the HbA1C from baseline to six months post-intervention was significantly better among the intervention group -0.94% (95% CI -1.46, -0.41) compared to the control group 0.73% (95% CI -0.09, 1.54) *p* < 0.001.

**Conclusion:**

Our study showed that among patients with insulin-dependent type 2 diabetes residing in rural Rwanda, SMBG was feasible and demonstrated positive outcomes in improving blood glucose control. However, there is need for strategies to enhance accuracy in recording blood glucose test results in the log-book.

**Trial registration:**

The trial was registered retrospectively on the Pan African Clinical Trial Registry, on 17^th^ May 2019. The registration number is PACTR201905538846394.

## Background

Currently, approximately 19 million adults in Africa suffer from diabetes mellitus (DM). Moreover, global projections indicate that, compared to other continents, Africa will experience the largest percentage increase of approximately 143% in the burden of DM by 2045 [1]. These estimates are presumed to largely be driven by DM in urban populations; however, DM has been endemic but inadequately studied in rural sub-Saharan Africa (SSA) [2].

While 60% of the population in SSA live in the rural regions, access to quality DM management in rural Africa remains a significant challenge [3]. Health care facilities are ill-equipped and lack adequate human resources, diagnostic and monitoring capacity, essential medicines and other effective strategies to manage diabetes [4–7]. This limited care may be associated with significant morbidity and life-threatening complications [8].

These challenges are especially true for insulin-dependent patients who require insulin dose adjustments and need to monitor potential life-threatening complications related to medication self-management. Specifically, clinicians primarily rely on one point of care random blood glucose measurements taken during clinical visits to inform insulin dose adjustments. Although the single random blood glucose may provide a patient’s glucose level at the time of the clinic visit, it is a poor measure for understanding the patient’s average blood glucose control. With limited glucose data, clinicians risk under or over prescribing insulin doses, thus increasing the risk of hypo- or hyperglycemic episodes among patients. Even with appropriate dose adjustments, transient or seasonal food insecurity in SSA may lead to hypoglycemia, which should be noted as early as possible through both recognition of symptoms and repeated blood glucose measurements.

Quality DM care in SSA requires proper and effective strategies to monitor and enhance glycemic control. Self-monitoring of blood glucose (SMBG)—shown to improve outcomes and enhance the quality of life among patients with DM—is a key component to achieving adequate glycemic control [9]. However, the overwhelming evidence on DM care comes from high income countries. The limited studies done in SSA on implementing SMBG included urban populations and indicated several challenges including but not restricted to poor knowledge of SMBG, high costs of test strips and glucose meters and suboptimal adherence and consistency among patients who receive SMBG education [10, 11].

## Methods

### Study aims

This study sought to assess the feasibility and effectiveness of implementing SMBG among insulin-dependent type 2 DM patients receiving care in three rural district hospitals in Rwanda.

### Study design

We conducted a six-month open randomized controlled trial, including patients diagnosed with type 2 DM taking insulin and being managed at outpatient non-communicable disease (NCD) clinics in three rural Rwandan district hospitals. This study adhered to CONSORT guidelines.

### Study setting

The study was conducted at the three public district level hospitals in rural Rwanda: Kirehe and Rwinkwavu district hospitals in the Eastern Province, and Butaro district hospital in the Northern Province. As described elsewhere [12], the three district hospitals are under the supervision of the Rwanda Ministry of Health (RMOH) and receive regular technical and financial support from Partners In Health/Inshuti Mu Buzima (PIH/IMB)—a non-governmental organization that has been supporting health care services in Rwanda since 2005.

In Rwanda, patients with insulin-dependent type 2 DM receive routine care at integrated NCD outpatient clinics, known as PEN-Plus. This nurse-led approach builds on the WHO PEN (Package of Essential Non-communicable diseases) model, which is primarily focused on common, non-severe NCDs, such as mild to moderate hypertension and non-insulin-dependent diabetes, at health centers [13]. PEN-Plus clinics are based at district hospitals and focus on the management of severe NCDs, such as insulin-dependent type 1 and 2 DM and heart failure [14]. Type 2 DM patients are referred to these clinics from in-patient hospital facilities, the general outpatient facility or from lower level facilities, such as health centers.

The management of type 2 DM patients in these clinics is guided by standardized national diabetes treatment protocols developed by the Rwandan MOH in collaboration with PIH/IMB and other partners. The criteria to initiate insulin medication among the diabetes patients is outlined in the treatment protocols [15].

### Study population

The study focused on patients with insulin-dependent type 2 DM and enrolled in PEN-Plus clinics at one of the three district hospitals.

### Inclusion and exclusion criteria

Adults aged at least 18 years of age diagnosed with insulin dependent type 2 DM and receiving an insulin regiment at the time of study at one of the three above listed district hospitals were eligible. Patients also had to demonstrate an ability to read or had a reliable person who could write sufficiently to record glucose level readings into study provided log-books. Eligible participants must have had the most recent HbA1C recording at 7% or greater.

Participants were excluded if they had a diagnosis of type 1 diabetes, gestational diabetes or chronic kidney disease or were unable to read and write sufficiently to use log-books and had no reliable person who would assist in reading the glucose levels and using the log-book.

### Enrollment of participants

Participants were identified through electronic medical records at the three district hospitals and those eligible were recruited and enrolled sequentially from April to June 2019. Study participants were followed monthly for a period of six months from their date of enrollment. Data collection occurred from April to December 2019.

### Sample size and power

All patients with insulin-dependent type 2 DM at the three hospitals who met the inclusion and exclusion criteria were included. From previous literature in this setting, we assumed that the change in HbA1C would be 0.2% among the control group [16] and 1.2% among the intervention group [11]. With a standard deviation of 1.9% or less, our eligible sample size of 82 study participants would yield a power of greater than 80% to detect the mean difference of the change in HbA1c among the intervention group compared to that of the control group.

### Randomization

A simple random allocation sequence for each of the three study sites was computer generated by the research associate who did not have access to patient’s clinical records and was not involved in patient enrollment. The research study coordinator, who was also responsible for the recruitment process conducted the allocation process using sealed envelopes. Participant distribution followed a 1:1 ratio between study groups.

### Intervention procedure

Both the intervention and control groups continued with their usual DM management, which consisted of routine monthly medical consultation and education. In addition, the intervention group was given an SMBG kit comprising of glucose meter machine, log-books, blood lancets and test strips to implement SMBG at home. All participants in the intervention group received training targeted towards integrating SMBG. The training topics included appropriate use of the SMBG kits and proper waste disposal mechanisms. Additionally, educators reviewed the signs and symptoms of hypo- and hyperglycemia and course of action in case the participants experienced such episodes. While the training on hypo- and hyperglycemia are part of the routine education curriculum, this SMBG education session integrated the use of glucose meters to inform self-management of such episodes. Participants were instructed to take their blood glucose levels every day of the week, conducting one test per day. The daily testing followed a schedule of alternating the time of day for testing. The set times included morning, midday, and evening (post-meal). Participants were also given a mobile phone number belonging to the study coordinator to communicate in-case of any concerns or questions related to implementing the SMBG.

The control group did not receive any additional changes to its day-to-day care.

### Outcome measures

The outcome measures were assessed at enrollment, three months and six months’ post-intervention. At enrollment, demographic and self-reported basic clinical measures were collected using a standard questionnaire. We collected demographic information and clinical variables including: sex, age, marital status, education level, occupation, body mass index, waist, circumference, duration since diagnosis with insulin, and insulin regimen used. The primary and secondary study outcomes are described as follows.

The primary outcome measures were: 1) the change in HbA1c which was performed using point of care devices (SD A1cCare) and required a lancet-induced drop of blood collected from the participant’s fingertip. The resulting percent value reflected the participant’s blood glucose level over the past 1–3 months. 2) Implementation feasibility outcomes including adherence to SMBG which was measured by the participant’s adherence to the use of glucose meter and recording DM readings into log-books while practicing SMBG. Adherence to SMBG was calculated by dividing the actual number of SMBG readings recorded on the patient’s glucose meter and log book records by the expected number of SMBG glucose readings at six months. Participants with proportions of 80% or above were considered to have good adherence to SMBG while those less than 80% were considered to have poor adherence.

A secondary implementation outcome was the reliability of log-books, which was measured among the intervention group by comparing what was recorded in the log-book to what was automatically recorded by the glucose meter machine. Lastly, mean blood glucose readings were calculated as the row means of the overall glucose meter readings for each of the study participants.

### Data analysis

Frequencies, percentages, means and standard deviations (SD), medians and interquartile range (IQR) were used to report continuous variables. A paired t-test was used to assess the change in HbA1c within the intervention and control group, while a two-sample t-test was used to assess the mean differences of HbA1c for the intervention group compared to the control group. We used the Wilcoxon rank sum test to test the association of demographic variables and the mean blood glucose for continuous variables and chi-square or fishers test for the categorical variables. Stata version 15 software was used to conduct the analysis.

## Results

### Demographic and clinical characteristics

**Fig. 1:**
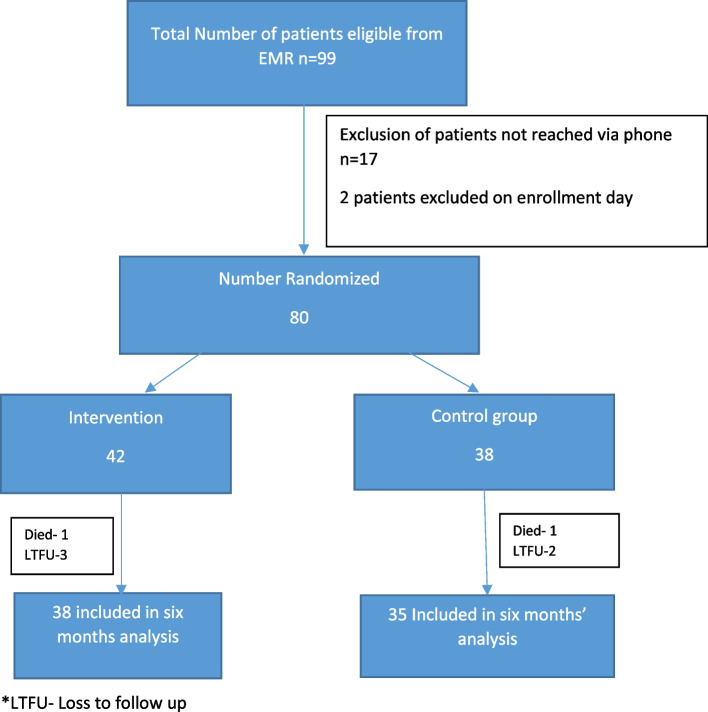
Number of patients screened and enrolled into the study

Of the 80 insulin-dependent type 2 DM patients included in this study, 42 were in the intervention arm and 38 in the control arm. By the sixth month of follow up three patients were lost to follow up on the intervention group while one died of hepatocellular carcinoma. In the Control group, two patients were lost to follow up while one died of complications related to diabetes foot and amputation (Fig. 1). In both the intervention and control arms, more than half of the participants were female (59.5% vs 52.6%) and the majority were married (71.4% vs 73.7%). Most of the participants had a primary level education or below (83.3% vs. 89.4%), and roughly half of participants were engaged in farming as a form of employment (54.8% vs. 50.0%). The participants’ age ranged from 30 to 88 years with an overall mean of 51.0 (SD ± 13.3) years (Table 1). For the intervention and control groups, the mean body mass index (BMI) was 23.92 (SD = 4.3) and 27.18 (SD = 10.2), respectively, and a median waist circumference of 90 cm (IQR: 84.0, 98.0) vs. 86 cm (IQR 83.0, 96.0) respectively. The median duration since the participants were diagnosed with type 2 diabetes was 8.6 years (IQR: 3.4, 14.1) for the intervention and 9.4 years (IQR: 6.3, 12.3) for the control group.

**Table 1:** Demographic and clinical characteristics of insulin dependent type 2 diabetes mellitus patients in Rwanda

Variables	Intervention	Control	*P*-value
	**Count (percentage)**	**Count(percentage)**	
***Gender***			
Female	25 (59.5)	20(52.6)	0.535
Male	17 (40.5)	18 (47.4)	
***Age years median (IQR)***	53.19 (41.32, 58.33)	50.38 (39.41, 62.44)	0.062
***Marital status***			
Single/widowed/ divorced /separated	12 (28.6)	10 (26.3)	0.821
Married	30 (71.4)	28 (73.7)	
***Education Level***			
Primary or Less	35 (83.3)	34 (89.4)	0.525
Secondary or Higher	7 (16.7)	4 (10.5)	
***Occupation***			
Farmer	23 (54.8)	19 (50.0)	0.287
Casual work	15 (35.7)	17 (44.7)	
Formal	3 (7.1)	0 (0.0)	
Missing	1 (2.4)	2(5.3)	
***BMI***			
Underweight	1 (2.4)	1 (2.6)	0.727
Normal	30 (71.4)	22 (57.9)	
Overweight/Obese	11 (26.2)	13 (34.2)	
Missing	0	2 (5.3)	
**Mean BMI (SD)**	23.92(4.3)	27.18 (10.2)	0.063
***Waist circumference cm, m(IQR)***	90 (84.0, 98.0)	86 (83, 96)	0.403
***Duration since diagnosis with diabetes years, m (IQR)***	8.62 (3.4, 14.1)	9.41 (6. 3, 12.3)	0.539
***Diabetes drug regimen***			
Insulin treatment only	16(38.1)	15 (39.5)	0.930
Insulin + oral drug	26 (61.9)	23 (60.5)	
**HbA1C at enrollment, mean (SD)**	7.96 (2.15)	7.48 (1.72)	0.278

The majority of study participants were being treated with insulin and oral medication as opposed to insulin only (61.9% vs 60.5%) and had a mean HbA1C of 7.96% (SD: 2.15) among the intervention group and 7.48% (SD: 1.72) among the control group (Table 1).

### Impact of SMBG

The mean difference in the HbA1C from baseline to six months’ post intervention for the intervention group was -0.94% (CI -1.46, -0.41), while that in the control group was 0.73% (CI -0.09, 1.54) (Table 2). Both the change in HbA1c for the intervention group and the difference between the intervention and control groups were statistically significant.

**Table 2:** Mean differences between HBA1C at 6 months and baseline within study arms

Study arm	Mean (SD) HbA1C at baseline	Mean (SD) HbA1C at six months	Mean (CI) difference^*^	*P*-value
**Intervention (*n* = 38)**	7.98 (1.86)	7.05(1.61)	-0.94 (-1.46, -0.41)	< 0.001
**Control (*n* = 35)**	7.26 (1.41)	7.99(2.38)	0.73 (-0.09, 1.54)	0.0794
			*p*-value comparing mean differences^+^	< 0.001

Note: Control, *n* = 38 at baseline and *n* = 35 at six months; 2 patients LTFU and 1 died; Intervention, *n* = 42 at baseline and *n* = 38 at six months; 3 patients LTFU and 1 died

^*^Statistic used: paired t-test

^+^two sample t-test

### Adherence to glucose meter and log-book use

Among those recruited into the intervention group, nearly two-thirds of the participants (63.4%) showed good adherence to performing the SMBG based on the number of tests recorded on the glucose-meters, however less than a third of the participants (29.3%) demonstrated good adherence based on the log-book data (Table 3).

**Table 3:** Glucose meter and logbook data

Glucose meter data	**Glucose-meter data**	**Log-book data**
	**Count (%)**	Count (%)
Adherence^*^		
> = 80% of expected blood glucose readings	26 (63.4)	12 (29.3)
< 80% of expected blood glucose readings	15 (36.6)	29 (70.3)
Level of mean blood glucose^**^		
> 180	24(58.5)	25 (61.0)
< = 180	17 (41.5)	16 (39.0)

^*^For the adherence we considered patients who had at-least 80% of the expected tests (roughly 180 tests); This is based on how many tests were done, but not whether they did them at the appropriate time

^**^Mean blood glucose: This is the overall mean (calculated as row mean of the reshaped dataset) of the glucose meter readings for each participant

The majority of participants had a mean blood glucose level greater than 180 mg/dl (58.5% based on the glucose meter readings and 61.0% from the log-book recordings) (Table 3).

### Reliability of log-book data

When comparing the dates, the time (± 1 h) and the blood glucose level data as recorded on the log-book records versus that recorded on the glucose meter machine, 55.3% of the log-book readings and those recorded on the glucose meters matched.

### Association of demographic characteristics, adherence to glucose meter use and mean blood glucose among the intervention group

Marital status was significantly associated with adherence to glucose meter. However, there was no significant association between adherence to glucose meter use and the other variables (Table 4).

**Table 4:** Association of demographic factors with mean blood glucose and adherence to glucose meter among the intervention group

Association between adherence and participants’ demographics			
	< 80% adherence to blood glucose testing	= > 80% adherence to blood glucose testing	
**Occupation**			
Farmer	9 (75.0)	11(55.0)	0.342
Professional	1 (8.3)	1(5.0)	
Casual	2 (16.7)	8(40.0)	
**Marital status**			
Married	12 (100)	13 (61.9)	0.030
Divorced/single/separated	0	8 (31.1)	
**Level of education**			
Primary or less	11 (91.7)	17 (80.9)	0.630
Secondary or higher	1 (8.33)	4 (19.1)	
**BMI**			
Normal	9 (75.0)	14 (66.7)	0.710
Obese/overweight	3(25.0)	7 (33.3)	
**Diabetes duration (continuous)**			0.801
**Age (continuous) *n* = 33**			0.539
**HbA1C at six months (continuous)**			0.714

## Discussion

In this study, after six months of implementing SMBG, participants showed significant improvement in their HbA1C levels compared to those who did not implement SMBG. Also among those in the intervention group, approximately two thirds showed good adherence to performing SMBG based on glucose meter data while less than a third demonstrated good adherence based on log-book data. In addition, slightly more than half of the data recorded on the log-book matched the records on the glucose-meters.

Consistent with the results from other studies conducted within East Africa [11, 17, 18], our results indicated an improvement of HbA1C level among the group that implemented SMBG. Unlike prior studies which included non-rural populations, our findings suggest that even with our completely rural population, which is challenged by implementation barriers including low literacy and low socio-economic status [19, 20], SMBG had a positive impact.

Our study findings on the adherence to SMBG, showed that nearly two thirds of the participants in the intervention group conducted at least 80% of the expected SMBG tests as recorded on the glucose meter. While these finding are still considered sub optimal, they are higher than those recorded in a study conducted in Kenya [11]. It is worth to note that participants who were on insulin regimen in the Kenyan study, performed SMBG test two to three times a day, on a daily basis. This difference in the testing schedule may explain the difference in the adherence levels. Other studies conducted in high income countries including approximately four tests per day have also shown sub-optimal adherence to SMBG [21, 22].

Despite the relatively higher adherence in our population, less than a third of the patients recorded at least 80% of the expected SMBG tests on the log-books. In addition, slightly more than half of the log-book recordings matched the glucose meter recordings. The non-matching recordings were characterized by differing dates, blood glucose level values or time of the day. Similar findings on inaccuracies on the records of log-book data compared to glucose meter data have been shown in other studies [23, 24]. Given the low recording rates and sub-optimal accuracy in the log-book records, we believe that reliance on log-book records alone may not be sufficient and may lead to inaccurate clinical decisions in the management of diabetes within our setting. While clinicians may opt to only review readings directly from glucose meters, the log-book interface allows clinicians to more easily identify patterns and trends to inform dose adjustments and patient education. If future adjustments to the patient education on log-book use does not improve adherence, then alternative strategies, such as direct downloads of the glucose meters’ readings to a digitized platform, should be explored. Other than marital status, our study showed no significant association between the participant’s demographic characteristics and adherence to SMBG. This association may reflect the value of home-based support to patients living with insulin-dependent type 2 DM. For this reason, our patient education sessions encourage participation of household members.

Our study had some limitations due to limited resources and a fixed SMBG schedule among the study participants. Only pre-prandial glucose readings were included, and we did not include post-prandial or reliable fasting blood glucose readings. However, we hope that insights provided from this study will inform follow up studies that include more testing resources to be able to test at other times, including post-prandial and bed-time blood glucose levels. In addition, our study did not assess other factors that could be associated with adherence to implementing SMBG and change in blood glucose levels. Other factors may include family support, food insecurity, and lifestyle factors, such as physical exercise and diet.

## Conclusion

To our knowledge, this is the first randomized trial assessing the effectiveness and feasibility of implementing SMBG in rural SSA. Our study showed that despite implementation barriers, especially around adherence to the use of log-book, SMBG was feasible to implement and was associated with significant improvement in their HbA1C levels. This may be partially due to instant feedback at home reminding patients of their glucose levels at different points of the day, which motivated them to eat better, adhere to medication regimens, or improve other lifestyle decisions. Future research, including our ongoing qualitative study on participants’ perspectives on the implementation of SMBG, can help elucidate this question. Future research may also help us to understand if clinicians altered dose adjustments more frequently or aggressively due to the additional blood glucose data points. Additionally, a longitudinal study design will enhance understanding of the long-term sustainability of the HbA1C changes. Finally, the findings from this study can be used as a reference point to develop contextually and culturally appropriate strategies to support SMBG in Rwanda and other similar settings in SSA.

## Data Availability

The dataset used for this research project may be available upon reasonable request from the Inshuti Mu Buzima research committee, Kigali, Rwanda. The request should be addressed to the chair of the committee who is also the research and training director at Inshuti Mu Buzima/Partners in Health Rwanda.

## Abbreviations

SSA: Sub Saharan Africa
SMBG: Self-monitoring of blood glucose
DM: Diabetes Mellitus
NCD: Non-communicable diseases
PIH/IMB: Partners In Health/Inshuti Mu Buzima
PEN: Package of Essential Non-communicable diseases
IQR: Interquartile range
SD: Standard Deviation
